# Clinical Impact of Angiographic Complications Occurring During Percutaneous Coronary Interventions

**DOI:** 10.1002/ccd.70515

**Published:** 2026-02-10

**Authors:** Emiliano Bianchini, Mattia Lunardi, Fabio Casamassima, Francesco Bianchini, Mattia Basile, Enrico Romagnoli, Cristina Aurigemma, Lazzaro Paraggio, Francesco Fracassi, Rocco Antonio Montone, Antonino Buffon, Luigi Cappannoli, Carolina Ierardi, Tommaso Sanna, Antonio Maria Leone, Carlo Trani, Francesco Burzotta

**Affiliations:** ^1^ Department of Cardiovascular Sciences Università Cattolica del Sacro Cuore Rome Italy; ^2^ Cardiology Department Galway University Hospital Galway Ireland; ^3^ Department of Cardiovascular Sciences Fondazione Policlinico Universitario A. Gemelli IRCCS Rome Italy; ^4^ Cardiology Department La Paz University Hospital Madrid Spain

**Keywords:** angiographic complications, PCI, prognosis

## Abstract

**Background:**

The clinical impact of different types of angiographic complications during elective PCIs remains largely unexplored.

**Aims:**

To explore the incidence, management, and outcomes of angiographic complications by type and severity during non‐urgent, non‐CTO PCIs.

**Methods:**

Clinical and procedural characteristics were prospectively collected and entered in a catheterization laboratory database, including a section dedicated to PCI complications, from 2015 to 2024. Angiographic complications were classified in eight categories: dissections, perforations, device entrapment/fracture, side branch flow impairment, vessel thrombosis, distal embolization, and any temporary or persistent slow‐flow or total occlusion. 30‐days and 1‐year all‐cause mortality were compared between two cohorts with or without angiographic complications, after a propensity score matching for demographic, clinical, procedural and anatomical characteristics.

**Results:**

Out of 8401 non‐urgent PCIs, 420 (5.0%) presented ≥ 1 angiographic complication (417 patients), with the following incidences: 2.2% iatrogenic dissections, 1.2% side branch flow impairment, 1.2% slow flow, 1.0% vessel occlusion, 0.4% distal embolization, 0.4% vessel thrombosis, 0.4% perforations, 0.1% device entrapment/fracture. Patients with angiographic complications showed a significantly higher risk of 30‐days mortality compared to the propensity score‐matched cohort without angiographic complications (4.1% vs. 1.6%, *p* = 0.045). Age, a previous hospitalization for decompensated heart failure, and a procedure on severely calcified lesions were independently associated with 1‐year mortality in patients with angiographic complications.

**Conclusion:**

Angiographic complications during elective PCI significantly increase 30‐day mortality compared with uncomplicated PCI. A history of decompensated heart failure warrants particular caution in the event of angiographic complications, as it is associated with an increased risk of mortality within the first year after the procedure.

**Abbreviations**
ACS: Acute Coronary Syndrome
AMI: acute myocardial infarction
AV: Atrio‐Ventricular
CAD: Coronary Artery Disease
CCS: Chronic Coronary Syndrome
CTO: Chronic Total Occlusions
DAP: Dose‐Area Product
DEB: Drug‐Eluting Balloons
DES: Drug‐Eluting Stents
IAPB: Intra‐Aortic Balloon Pump
IVI: Intravascular Imaging
LAD: Left Anterior Descending
LCX: left circumflex
LM: Left Main
NHLBI: National Heart, Lung, and Blood Institute
OCT: Optical Coherence Tomography
PAEs: Procedural Adverse Events
PCI: Percutaneous Coronary Intervention
RCA: right coronary artery
SB: Side Branch
TIMI: Thrombolysis in Myocardial Infarction

## 1 Introduction

Elective percutaneous coronary intervention (PCI) represents a pillar in the management of patients with coronary artery disease (CAD) [1]. Despite significant advancements in PCI devices and techniques, intraprocedural complications still occur and are promptly recognized by angiography; therefore they are usually referred to as “angiographic complications.” Such complications have a wide range of types and definitions but once occurred, have the potential to adversely impact clinical outcomes [2, 3].

Prior studies have extensively investigated the “no‐reflow” phenomenon and different thrombo‐embolic complications arising from urgent PCIs performed in the setting of acute coronary syndromes (ACS) [4, 5, 6]. On the other hand, data regarding the incidence and prognostic impact of different type of angiographic complications—both mechanical and thromboembolic—in the context of elective PCIs, are surprisingly scarce and difficult to be compared due to high variability of reporting and management [7]. In the past years, expert operators treating patients with chronic total occlusions (CTO) proposed classifications and management algorithms for angiographic complications [8]. For instance, these proposals are largely derived from personal experience rather than robust observational data [9]. To address this gap in knowledge, we conducted the present study analyzing the incidence, management, and outcomes of angiographic complications by type and severity during non‐urgent, non‐CTO PCIs in the era of second‐generation drug‐eluting stents (DES).

## 2 Methods

### 2.1 Study Objectives

The objectives of the present study is to describe the incidence and clinical impact of angiographic complications occurring during contemporary non‐urgent PCIs.

### 2.2 Study Design

This is an observational, single‐center analysis of prospectively collected data from consecutively treated patients at our Institution, where the clinical outcome of patients presenting ischemic heart disease undergoing coronary revascularization is continuously monitored within the Institutional clinical management pathways dedicated to patients with different coronary syndromes. The study followed the STROBE reporting guidelines. At our Institution, the clinical and procedural data of all patients afferent to the catheterization laboratory are prospectively entered into a dedicated structured database by the operator after the completion of the procedure using Suitestensa PACS software (Esaote©). This database was previously employed to assess the impact of surgical scores on PCIs and the safety of trans‐radial procedures [10, 11].

The initial screening of the study population included all patients undergoing PCI at our Institution since the introduction of latest generation DES (January 2015) to March 2024, and the inclusion criteria were:

- Presence of at least one angiographic complication as reported in the catheterization laboratory database;
- non‐urgent procedures (elective procedures for chronic coronary syndrome [CCS] or staged interventions on stabilized ACS);
- age > 18 years.

Patients or the public were not involved in the design, conduct, reporting, or dissemination plans of our research.

### 2.3 Data Extraction

All the procedural data were extracted from the Institutional electronic database of PCIs cited above.

Additional clinical variables, along with in‐hospital stay details, and discharge letters, were extracted from the electronic general hospital database. Survival at 1‐year was collected for all patients from a regional database, which monitors patients' survival status. A dedicated event adjudication committee (EB, MB, FC) verified and confirmed all the events reported, while remaining unaware of the procedural outcomes, including the occurrence and type of angiographic complications. Missing data did not exceed 5% for each variable included in the study. The study complied with the Declaration of Helsinki, and all patients signed an informed consent to the PCI procedure that included the use of collected data for scientific analyses. The local Ethics Committee approved the use of these data for the present retrospective study.

### 2.4 Definitions

#### 2.4.1 Angiographic Complications

All procedure records and angiographic images were reviewed by two expert interventional cardiologists (ML, ER) unaware of the clinical history and the clinical outcomes of the patients.

Angiographic complications were categorized into the following eight distinct groups:

- Iatrogenic dissections, classified according to the National Heart, Lung, and Blood Institute (NHLBI) classification; Non–flow‐limiting dissections occurring immediately after pre‐dilation—but prior to stent deployment—were classified as angiographic complications only if they extended into at least two additional contiguous segments adjacent to the one containing the treated lesion, thereby necessitating the implantation of a stent significantly longer than originally planned.
- Perforations, classified using the Ellis classification [12].
- SB flow deterioration: a temporary or persistent Thrombolysis in myocardial infarction (TIMI) flow grade 0–2 in a SB vessel measuring ≥ 1 mm in diameter, either stemming from the target lesion or located within 5 mm of it. This definition aligns with a previous study on SB occlusions during bifurcation PCIs, showing that patients with a side‐branch ≥ 1 occlusion was related to worse 1‐year outcomes [13].
- Operative vessel occlusion: temporary or persistent TIMI 0 flow in the treated vessel.
- Distal embolization: a temporary or persistent occlusion in any distal segment of the treated vessel caused by one of the following suspected or confirmed mechanisms: embolization of iatrogenic materials (e.g., following balloon rupture), thrombus embolization, or disrupted plaque material embolization with or without slow flow (TIMI 1–2) in the operative vessel [14].
- Operative vessel slow‐flow: any temporary or persistent slow flow (TIMI 1–2) during the procedure.
- Vessel thrombosis: any angiographic haziness suggestive of acute thrombus formation.

Further details on angiographic definitions adopted in the study are reported in Supporting Information S1: Method 1.

#### 2.4.2 Endpoints

The primary endpoint of the study was all‐cause death at 1‐year. The survival rate of patients with angiographic complications was compared with that of a propensity score–matched cohort of patients without angiographic complications derived from the same institutional PCI database.

The main secondary endpoints were final angiographic success (TIMI flow 3 in all vessels), procedural adverse events (PAEs) and 30‐days all‐cause death after the detection of the angiographic complication.

PAE was defined as the composite of:

- procedural arrhythmias (bradyarrhythmia or tachyarrhythmia) requiring direct current shock, cardiopulmonary resuscitation, or urgent temporary pacing;
- hemodynamic instability, defined as a mean arterial blood pressure drop below 65 mmHg (with normal baseline values) requiring the use of inotropes/vasopressors and/or mechanical circulatory support;
- the need for emergency surgery.

Events were reported for the entire cohort of patients who experienced at least one angiographic complication, as well as for subgroups of patients with specific types of complications. Since patients could experience multiple angiographic complications during the same procedure, subgroup assignment was based on the first complication that occurred. This approach was adopted for two key reasons: (1) multiple angiographic complications are often secondary to a single angiographic complication (e.g iatrogenic dissection leading to vessel occlusion; device entrapment leading to a iatrogenic dissection); (2) restricting the analysis to procedures with a single angiographic complication would introduce a significant selection bias and reduced statistical power. An UpSet plot was used to visually represent the most common overlaps among different types of angiographic complications, as well as the frequency of subsequent cascade complications following the initial complication.

### 2.5 Statistical Analysis

Continuous variables were tested for identifying normal distributions according to the Shapiro‐Wilk test and Q‐Q plot visual examination. They were compared with an unpaired Student *t* test or with the Mann‐Whitney U nonparametric test, when appropriate. Data were expressed as means ± standard deviation (SD) or median and inter‐quartile range (IQR). Categorical variables were compared using χ2 or Fisher's exact test as appropriate and expressed as frequencies and percentages. Since patients could exhibit multiple angiographic complications during the procedure, an UpSet plot was generated to illustrate which complications most frequently occurred together. In order to compare the prognosis of patients with angiographic complications with a cohort without angiographic complications from the Institutional PCI database, a one‐to‐one PS matching was used with the nearest‐neighbor approach using a calliper method, with calliper set to 0.2. The absolute standardized mean difference (SMD) for all the variables of interest was calculated to report the balance between the matched cohorts, with a value below 0.2 considered as acceptable. All the variables used for PS‐matching are reported in Supporting Information S1: Method 2.

Survival curves of the PS‐matched groups with and without complications and of different subgroups with specific angiographic complications were constructed with the use of Kaplan‐Meier estimates, and the Log‐Rank test was performed to test any significant difference. Clinical follow‐up was censored at the date of clinical event or latest available follow‐up. Univariate Cox proportional hazards regression was performed for all the variables collected to find predictors of 1‐year all‐cause mortality in patients with angiographic complications. All the variables resulting as significant predictors of 1‐year all‐cause mortality (*p* < 0.05) were included in a multivariable Cox proportional hazard model to find the independent predictors. A sensitivity analysis using the least absolute shrinkage and selection operator (LASSO) regression was performed to refine candidate variables selection and increase the event‐per‐variable ratio in the multivariable model: the penalty parameter (λ) was chosen via 10‐fold cross‐validation to minimize prediction error, and only variables with non‐zero coefficients in the optimal model were retained for inclusion in the final multivariable cox regression model. To test multicollinearity of the predictive models, the variance inflation factor was calculated, and if a significant degree of correlation was detected between variables composing the model, the one with the lowest clinical relevance was excluded. All data analyses were performed using SPSS version 29.0 (IBM Corp, Armonk, NY, USA) and STATANoW version 18.0 (Stata Corporation LLC, College Station, TX, USA).

## 3 Results

### 3.1 Study Population

**Figure 1:**
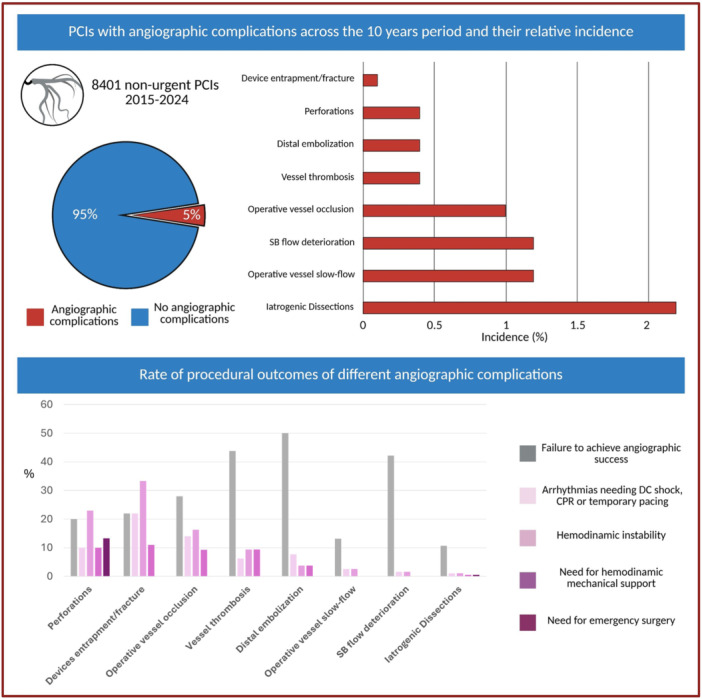
Percentage of PCIs with angiographic complications across the 10 years period and their relative rate of procedural adverse events. CPR, cardio‐pulmonary resuscitation; DC, direct current; PAE, procedural adverse event. PCI, percutaneous coronary intervention; SB, side branch. [Color figure can be viewed at wileyonlinelibrary.com]

Out of 10,692 PCIs performed between January 2015 and March 2024, 8401 were non‐urgent and involved non‐CTO lesions. A total of 545 procedures were reported by the operator to have at least one angiographic complication. Of these, 125 procedures were excluded as the complication reported was not angiographically documented or did not meet the definitions for angiographic complications adopted in the present study—mostly because minor iatrogenic dissections after lesion pre‐dilation were reported. Thus, the final study cohort included 420 procedures—among 417 patients experiencing at least one procedure with angiographic complications (5.0% of elective PCIs, Supporting Information S1: Figure 1).

The types of angiographic complications are reported in Figure 1. Over 8401 procedures, angiographic complications presented the following rate:

- 189 iatrogenic coronary dissections (2.2%)
- 98 side branch flow deteriorations (1.2%)
- 30 perforations (0.4%)
- 34 vessel thrombosis (0.4%)
- 83 vessel occlusions (1%)
- 96 slow flow (1.2%)
- 36 distal embolization of thrombus, plaque material, or device material (0.4%)
- Nine device entrapments, fractures, or crushes (0.1%)

**Figure 2:**
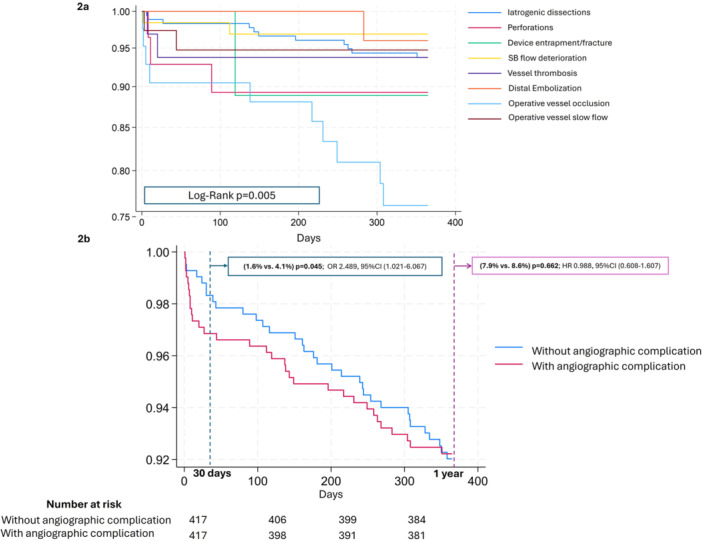
Kaplan‐Meier survival curves with Log‐Rank tests. 2a. Kaplan‐Meier survival curves with Log‐Rank test for different subgroups of patients with different first‐occurring angiographic complications. 2b. Kaplan‐Meier survival curves with Log‐Rank test between PS‐matched patients with or without angiographic complications. PS, propensity score; SB, side branch. [Color figure can be viewed at wileyonlinelibrary.com]

The UpSet plot in Supporting Information S1: Figure 2a illustrates the number of procedures in which more than one angiographic complication occurred. Supporting Information S1: Figure 2b illustrates the number of first angiographic complication for each category and the rate of subsequent cascade additional complications.

### 3.2 Baseline and CAD Characteristics

The mean age of the population was 70.8 ± 10.3 years, and 72.4% of the patients were male (Table 1). The mean Logistic EuroSCORE was 3.4 ± 4.2. The most frequent clinical presentation leading to PCI was CCS (85.1%). The remaining 14.9% of patients underwent staged PCI after an ACS, 7 (3–10) and the median waiting time from ACS diagnosis to PCI was 7 days (IQR 3–10). Most of patients presented single‐vessel (42.5%) or two‐vessel CAD (36.2%) and the median Jeopardy score was 9 (3–12). The vast majority of the procedures (87.5%) were performed on de novo lesion of native coronary arteries. Bifurcations were involved in 41.9% of the lesions, while 24.5% of procedures involved severely calcified lesions. Of note, 50% of lesions interested by perforations showed severe calcification.

**Table 1:** Population characteristics before the procedure and univariate Cox proportional hazard regression for 1‐year death (*N* = 417).

Variable		HR with 95% CI	*p* value
Age	70.8 ± 10.3	1.069 (1.031–1.110)	< 0.001
Sex (male)	304 (72.4)	0.713 (0.460–1.105)	0.131
BMI > 30	51 (12.1)	0.738 (0.320–1.702)	0.476
Hypertension	320 (76.7)	0.735 (0.445–1.213)	0.228
Diabetes	129 (29.9)	0.911 (0.490–1.693)	0.768
On oral medications	77 (18.4)	1.574 (0.878–2.821)	0.128
On insulin therapy	48 (11.4)		
Current smoker	62 (14.8)	0.775 (0.372–1.617)	0.497
Former smoker	91 (21.8)	1.195 (0.562–2.540)	0.644
Dyslipidemia	258 (61.8)	0.948 (0.485–1.852)	0.875
Family history of CAD	84 (20.1)	0.339 (0.104–1.105)	0.073
Creatinine > 1.2 mg/dL	21 (5)	1.836 (0.563–5.986)	0.314
Dialysis	9 (2.1)	3.104 (0.746–2.924)	0.120
Logistic EuroSCORE	3.4 ± 4.2	1.073 (1.024–1.124)	0.003
Prior MI ( > 3 months)	37 (8.8)	0.582 (0.140–2.423)	0.457
Prior PCI	140 (33.6)	0.462 (0.202–1.055)	0.067
Prior CABG	39 (9.3)	0.107 (0.003–3.314)	0.202
Prior HF hospitalization	40 (9.5)	2.622 (1.565–4.392)	< 0.001
Peripheral artery disease	20 (4.8)	2.754 (0.974–7.791)	0.056
Carotid artery disease	22 (5.2)	2.403 (0.850–6.794)	0.098
Prior TIA/stroke	7 (1.7)	1.643 (0.225–11.991)	0.625
Clinical presentation:		1.706 (0.778–3.744)	0.183
CCS	355 (85.1)		
Staged PCI after ACS	62 (14.9)		
Time from ACS (days)	7 (3–10)		
Single‐vessel disease	177 (42.5)	0.533 (0.257–1.105)	0.091
Two‐vessel disease	151 (36.2)	0.986 (0.499–1.946)	0.967
Three vessel disease	89 (21.3)	1.827 (0.899–3.714)	0.096
Jeopardy score	9 (3–12)	1.114 (0.989–1.254)	0.077

*Note*: Values are n (%), mean ± SD or median (interquartile range).

Abbreviations: ACS, acute coronary syndrome; CABG, coronary artery bypass grafting; CAD, coronary artery disease; CCS, chronic coronary syndrome; HF, heart failure; HR, hazard ratio; LAD, left anterior descending; LCX, left circumflex; LM, left main; MI, myocardial infarction; PCI, percutaneous coronary intervention; RCA, right coronary artery; SVG, saphenous vein graft; TIA, transient ischemic attack.

### 3.3 Procedural Characteristics

The main procedural details are reported in Table 2. LAD was the most frequent vessel treated (58.3%), and radial access, in keeping with the local expertise of the center, was used in almost all procedures (95.7%). The implementation of calcium modification/calcium debulking techniques was deemed necessary in respectively 2.3% and 2.9% of cases. IVI guided the procedure in 10.0% of cases, mostly through OCT (92.9%). In 5.3% of procedures, a high‐risk of procedural hemodynamic instability was anticipated so that a percutaneous support device (intra‐aortic balloon pump or Impella) was placed upfront.

**Table 2:** Lesion and procedural characteristics and univariate cox proportional hazard regression for 1‐year death (*N* = 420).

Variable		HR with 95% CI	*p* value
Severely calcified lesion	103 (24.5)	2.150 (1.353–3.415)	0.001
Bifurcation lesion	175 (41.8)	0.683 (0.349–1.339)	0.268
In‐stent restenosis	52 (12.5)	0.617 (0.189–2.011)	0.423
Percentage stenosis of the lesion related to the complication	82.1 ± 14.2	1.018 (0.988–1.049)	0.241
LM PCI	34 (8.1)	2.559 (1.468–4.461)	0.001
LAD PCI	245 (58.3)	0.803 (0.418–	0.512
LCX PCI	122 (29)	1.546)	0.179
RCA PCI	77 (18.3)	1.583 (0.810–3.095)	0.896
VGS PCI	9 (2.1)	1.057 (0.463–2.412)	0.529
Radial access use	402 (95.7)	0.552 (0.271–1.121)	0.100
Use of DEB	178 (42.5)	1.363 (0.709–2.619)	0.353
Use of DES	371 (88.3)	2.328 (0.559–9.689)	0.246
Stent diameter	3 (3–3)	0.920 (0.542–1.559)	0.756
Stent length	30 (21–38)	0.995 (0.964–1.027)	0.767
Use of cutting/scoring balloon	2 (0.5)	—	—
Use of intravascular lithotripsy	10 (2.4)	1.162 (0.159–8.480)	0.883
Use of calcium debulking devices	12 (2.9)	3.586 (1.099–11.695)	0.034
Use of IVI	42 (10)	0.254 (0.035–1.852)	0.176
OCT	39 (92.9)		
IVUS	3 (7.1)		
Use of upfront IAPB/Impella	22 (5.3)	3.828 (2.195–6.675)	< 0.001

*Note*: Values are n (%), mean ± SD or median (interquartile range).

Abbreviations: CPR, cardio‐pulmonary resuscitation; DAP, Total dose‐area product; DC, direct current; DEB, drug‐eluting balloon; DES, drug‐eluting stent; IVI, intravascular imaging; IAPB, intra‐aortic balloon pump; IVUS, intravascular ultrasound; OCT, optical coherence tomography; PAE, procedural adverse events; VF/SVT, ventricular fibrillation/sustained ventricular tachycardia.

### 3.4 Characteristics and Management of Angiographic Complications

Details about the mechanisms, subtypes, and site of each complication are reported in Supporting Information S1: Table 1. The majority of iatrogenic dissections, slow‐flow, SB flow deterioration, distal embolization, and vessel thrombosis occurred at the time of stent post‐dilation (respectively: 46.7%, 43.7%, 46.9%, 50.0%, and 58.8%). The occlusion of treated vessel occurred more frequently during the lesion preparation with balloon pre‐dilation (33.7%). Balloon pre‐dilation was also the most common mechanism causing perforations (56.6%), followed by distal perforations with guidewires (26.6%) and perforations at the edges of the deployed stents (16.6%). The most common type of device entrapment was the distal tip of “jailed” guidewires beneath the stents (33.3%), with subsequent fracture and damaged devices left in the coronary circulation. Although vessel occlusion may arise from different triggering mechanisms (e.g., iatrogenic dissection or thrombosis), in 43 cases vessel occlusion was the first angiographic event and was therefore considered the first‐occurring complication, as a single specific underlying mechanism could not be clearly identified (Supporting Information S1: Figure 2b).

Supporting Information S1: Figure 3 depicts the most common procedural strategies implemented to address the complications.

### 3.5 Procedural Outcomes

The overall rate of angiographic success was 76%. The failure to achieve angiographic success was highly variable across different complication types ranging from 10.7% after iatrogenic dissections to 50% after distal embolization (Figure 1).

The incidence of PAE considerably differed across the spectrum of angiographic complications, ranging from 1.1% for iatrogenic dissections to 44.4% for device entrapment/fracture (Table 3). Vessel thrombosis, vessel occlusion, perforations, and device entrapment/fracture were associated with the highest absolute rate of PAE (respectively: 15.6%, 25.6%, 26.6%, and 44.4%).

**Table 3:** Procedural outcome and survival data.

Procedural Outcomes	
Total DAP	32,876 (13,390–40,496)
Contrast dye (mL)	269 ± 117
Procedural time (min)	89 ± 46
Angiographic success	319 (76)
PAE	37 (8.8)
PAE components	
Any arrhythmias requiring DC shock, CPR or temporary pacing	19 (4.5)
Hemodynamic instability	24 (5.7)
Need for hemodynamic mechanical support	13 (3.0)
Need for emergency open surgery	5 (1.2)
Survival data	
Peri‐procedural death	4 (1.0)
30‐days death	17 (4.1)
1‐year death	36 (8.6)

*Note*: Values are n (%), mean ± SD or median (interquartile range).

Abbreviations: CPR, cardio‐pulmonary resuscitation; DAP, Total dose‐area product; DC, direct current; PAE, procedural adverse events.

### 3.6 Clinical Outcomes, PS‐Matching Analysis, and Predictors of 1‐Year Death

Among the subgroups of patients experiencing different first‐occurring angiographic complications, 1‐year survival differed significantly among them (log‐rank test *p* = 0.005; Figure 2a). Patients with operative vessel occlusion, perforation, or device entrapment/fracture had the highest absolute 1‐year mortality rates (25.6%, 16.0%, and 11.1%, respectively), (Supporting Information S1: Table 3).

After PS‐matching, 417 patients with angiographic complications were compared with 417 patients without angiographic complications. Clinical, procedural, and anatomical characteristics were similar between the two cohorts, and the standardized mean difference did not exceed 0.15 for any variable. Supporting Information S1: Table 2 reports the characteristics of the two cohorts and the corresponding standardized mean differences.

The survival rates at 1 year did not differ between the two cohorts (log‐rank test *p* = 0.662), but patients with angiographic complications exhibited a significantly higher 30‐days mortality (4.1% vs. 1.6%, OR 2.489, 95% CI 1.021–6.067, *p* = 0.045), (Figure 2b).

Two secondary survival analyses were performed for patients achieving a complete angiographic success or not at the end of the PCI, and for patients experiencing or not a subsequent PAE after the angiographic complication. Patients who achieved a final angiographic success did not show a significant difference in 1‐year survival compared to patients who did not (Log‐Rank test *p* = 0.144; Supporting Information S1: Figure 4a). On the opposite, patients with a PAE after the occurrence of an angiographic complication showed a worst 1‐year survival compared to patients without (Log‐Rank test *p* = 0.015; Supporting Information S1: Figure 4b).

Table 4 resumes the univariate and multivariate cox regression analysis for the prediction of 1‐year mortality. On multivariable Cox regression analysis, patient's age (HR 1.055, 95% CI 1.007–1.105, *p* = 0.024), a history of previous hospitalization for decompensated heart failure (HR 1.836, 95% CI 1.009–3.339, *p* = 0.047) or the presence of a severely calcified lesion (HR 1.706, 95% CI 1.025–2.839, *p* = 0.040) were independently associated to 1‐year death. Results were consistent in the more parsimonious multivariable model after the use of LASSO regression for variable selection. No significant multicollinearity by linear regression analysis was found in the multivariable models.

**Table 4:** Univariable and multivariable Cox regression analysis for the prediction of 1‐year death in patients with ≥ 1 angiographic complication during PCI.

Variable	Univariate analysis		Multivariable analysis		Multivariable analysis after LASSO regression selection	
	HR with 95% CI	*p* value	HR with 95% CI	*p* value	HR with 95% CI	*p* value
Age (years)	1.069 (1.031–1.110)	< 0.001	1.055 (1.007–1.105)	0.024	1.057 (1.018–1.097)	0.004
Prior HF hospitalization	2.622 (1.565–4.392)	< 0.001	1.836 (1.009–3.339)	0.047	1.865 (1.034–3.362)	0.038
Logistic EUROscore	1.073 (1.024–1.124)	0.003	1.005 (0.933–1.083)	0.896	—	—
Severely calcified lesion	2.150 (1.353–3.415)	0.001	1.706 (1.025–2.839)	0.040	1.778 (1.099–2.878)	0.019
LM PCI	2.559 (1.468–4.461)	0.001	1.496 (0.801–2.792)	0.206	1.482 (0.792–2.775)	0.218
Use of upfront IAPB/Impella	3.828 (2.195–6.675)	< 0.001	1.870 (0.948–3.690)	0.071	1.880 (0.959–3.686)	0.066
Use of Calcium Debulking device	3.586 (1.099–11.695)	0.034	1.337 (0.548–3.258)	0.523	—	—

Abbreviations: CI, confidence interval; HF, heart failure; HR, hazard ratio; IAPB, intra‐aortic balloon pump; LM, left main; PCI, percutaneous coronary intervention.

## 4 Discussion

The main findings of the study are:

1. Angiographic complications remain common in the contemporary era of PCI and specific types are associated to different rates of PAE and mortality.
2. Patients with angiographic complications had a significantly higher 30‐day mortality compared to patients without angiographic complications, despite similar demographic, clinical, procedural, and anatomical characteristics.
3. Patient's clinical characteristics such as a history of previous hospitalization for heart failure independently impact 1‐year prognosis in this subgroup of patients.

Previous studies focused on specific complications and were conducted more than a decade ago, when using old devices with proven worse performance [12, 15, 16, 17, 18, 19]. More recent investigations focused on CTO procedures, where the procedural and technical setting are completely different from our scenario [8, 9]. Moreover, this study reports data on non‐urgent PCIs, differently from all the previous studies including patients with acute myocardial infarctions (AMI), which might inherently bias the reporting of prognostic data for such complications [3, 12, 15, 20].

In terms of mechanisms, most of angiographic complications occurred during the post‐dilation of an already deployed DES (Supporting Information S1: Table 1). The pathophysiology lies in the dissections at the stent edges, excessive compression of soft plaques beneath the stent struts, and subsequent debris embolization or procedural thrombus formation [21, 22]. This was probably due to repetitive attempts of optimizing under expanded stents with high‐pressure inflations, when the target lesion was not optimally prepared. The high frequency of thrombotic and embolic events in this cohort of elective patients likely reflects the complex case mix of our referral center and earlier practice patterns with less intravascular imaging guidance. Additionally, our definition of embolization included plaque and device‐related debris, not only thrombus.

The use of calcium modification/debulking was surprisingly low in patients who experienced an angiographic complications, accounting for 5.4%, despite the high prevalence of severely calcified lesions (24.6%). The use of IVI—which was generally underutilized in this population—could have enhanced the accuracy of post‐dilation balloon sizing and provided valuable insights into the actual need for further post‐dilation and the risk of plaque material embolization or in‐stent protrusions [23]. Even more importantly, IVI may have enabled a more accurate assessment of plaque composition—particularly in severely calcified lesions—and guided appropriate lesion modification before aggressive pre‐dilation or stent implantation. Along these lines, the acute vessel occlusions and perforations were frequently observed following the pre‐dilation phase (Supporting Information S1: Table 1). A stepwise approach to the preparation of calcified lesions is worthwhile not only to optimize stent expansion but also to prevent such avoidable complications [24]. In a large‐scale sub‐analysis of the HORIZONS‐AMI (Harmonizing Outcomes With Revascularization and Stents in Acute Myocardial Infarction) and ACUITY (Acute Catheterization and Urgent Intervention Triage Strategy) trials, severe lesion calcifications by angiography definition were associated to abrupt vessel closure and long‐term prognosis [25]. These results are consistent with our findings and can be explained by two main factors. First, the presence of a severely calcified lesion inherently carries a higher risk of suboptimal PCI results if the lesion is not properly prepared, thereby increasing the likelihood of adverse outcomes. Second, 50% of perforations—one of the most clinically relevant angiographic complications in terms of mortality risk—occurred in severely calcified lesions. The univariate association of calcium debulking devices with 1‐year mortality reflects this concept, indicating a subset of lesions that were intrinsically more complex and biologically unfavorable. In fact, bearing in mind the limited size of this subgroup of patients, the use of calcium debulking techniques—intended as better lesion preparation—did not improve outcomes. In our cohort, among the 42 procedures performed with IVI guidance, only one patient (2.3%) died within 1 year, compared with 35 of 377 patients (9.3%) in the non‐IVI cohort. Because only a single event occurred in the IVI subgroup, no reliable statistical testing could be performed, but these findings may reflect either the ability of IVI to address the mechanistic causes of angiographic complications or its role in optimizing the final procedural result once the complication has been managed.

Anatomical and procedural complexity, together with patient frailty, are critical determinants of PCI outcomes and subsequent 1‐year mortality. This is well reflected in our PS–matched analysis: the survival curves of the two subgroups diverged sharply within the first weeks after the procedure, then gradually converged between 6 and 12 months (Figure 2b), when factors other than the complication itself began to predominate. In particular, both groups consisted of patients with a mean age above 70 years and with substantial baseline cardiovascular risk, including multivessel coronary disease in more than half of cases, and a history of prior PCI in over one‐third of patients. These characteristics likely reflected either a frail patient, more prone to worse prognosis in case of complications, or a heightened susceptibility to non‐cardiac events that increasingly influence prognosis after the early post‐procedural phase. Moreover, patients who survived the short‐term consequences of the complication were younger and likely less frail than those who died, which may have conferred a higher long‐term survival compared with the propensity‐matched non‐complicated cohort.

To our knowledge, this is the first study to report the survival patterns of these subgroups, highlighting the significantly higher short‐term mortality risk associated with angiographic complications compared with patients without such complications, independent of clinical or procedural complexity (e.g., older age, multiple prior interventions, multivessel disease, left main PCI, bifurcation PCI).

Adding to this context, since patients achieving complete TIMI 3 flow in all vessels at the end of the procedure did not demonstrate a significant better survival compared with those with different degrees of angiographic failure (Supporting Information S1: Figure 4a), clinical management should not be de‐escalated solely on the basis of the final TIMI flow. As further illustrated in Supporting Information S1: Figure 4b, the additional occurrence of PAEs may more accurately capture both the intrinsic severity of the angiographic complication and the patient's underlying clinical frailty—two factors that can influence prognosis despite the restoration of optimal flow as the final angiographic result.

Among the variables linked to worse 1‐year survival in patients with angiographic complications, a prior diagnosis of heart failure is particularly noteworthy, as it may help explain the increased mortality risk and guide refinements in patient monitoring and management. According to a Second Randomized Evaluation in PCI Linking Angiomax to Reduced Clinical Events (REPLACE‐2) sub‐study, the occurrence of most of the angiographic complications carries the risk of peri‐procedural myocardial infarction [17]. Myocardial loss following such complications may be particularly relevant in patients with pre‐existing heart failure, as their myocardium is already compromised, reducing their ability to tolerate additional injury and increasing the risk of worsening heart failure within 1 year of follow‐up. Unfortunately, in our registry, data on pre‐PCI and post‐PCI troponin levels, as well as complete echocardiographic assessments, were not consistently available. This limitation hindered the ability to explore the link between clinically diagnosed heart failure and post‐procedural changes in regional wall motion or global ejection fraction.

Finally, it is worth noting that evidence regarding the impact of standardized management strategies are limited to expert opinions and this is reflected by the number of different pharmacological and technical solutions adopted in our registry (Supporting Information S1: Figure 3) [26, 27]. Again, in the context of complications involving the damage of downstream coronary microcirculation or abnormal thrombus formation, studies are conflicting regarding the clinical benefit of a strategy over another, and are particularly scarce for PCI in patients with CCS [4, 28, 29].

## 5 Limitations

These results should be interpreted in light of several limitations. First, the data were derived from a single high‐volume tertiary care center, which may limit the generalizability of the findings to other settings—particularly to smaller or larger institutions with different patient populations and levels of operator experience. Furthermore, operator expertise accumulated in a high‐volume single center—together with the availability of advanced bail‐out techniques and robust intensive‐care resources—may not be representative of other settings, thus further constraining the external generalizability of these findings. Contrarily, the presence of numerous operators might have introduced inter‐operator variability in procedural steps and decision‐making, limiting the standardization of such procedures. Angiographic complications represent a heterogeneous group of events that often coexist within the same procedure and are typically managed based on the operator's individual judgment and experience. Although the propensity score was computed using more than 20 variables, unaccounted factors might still be present and influence the clinical outcomes of patients in the two subgroups considered. As detailed in the Methods section, vital status was reliably obtained through a regional survival registry but cause‐specific mortality could not be consistently adjudicated in approximately 40% of patients. To minimize the risk of misclassification and selective reporting bias, the analysis focused on all‐cause mortality only. Complete data on echocardiography, laboratory tests and post‐discharge therapy could not be implemented in our registry despite their clinical relevance (Central Illustration 1).

**Central Illustration 1:**
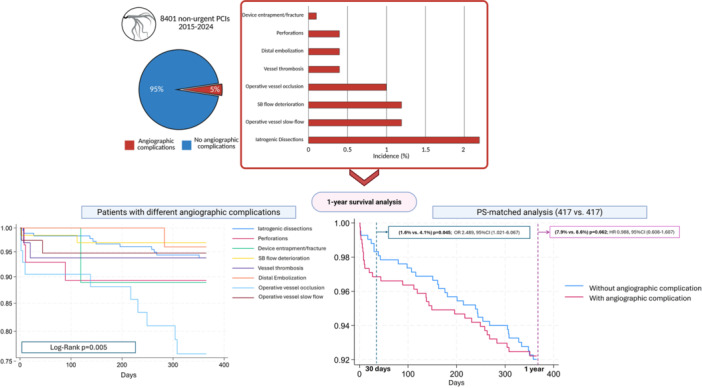
Incidence of different angiographic complications and 1‐year survival curves for different types of complications and for two propensity score‐matched cohorts with or without angiographic complications. [Color figure can be viewed at wileyonlinelibrary.com]

## 6 Conclusions

Despite advancements in PCI techniques, angiographic complications have still a tangible impact on short‐term prognosis, warranting a structured reporting in the catheterization laboratory; patients with heart failure require closer monitoring and tailored follow‐up, as they represent a particularly high‐risk subgroup.

## Funding

The authors received no specific funding for this work.

## Conflicts of Interest

F. Bianchini received a research grant from Abbott. E. Romagnoli received speaker fees from Abbott Vascular and Terumo. Aurigemma received speaker fees from Abbott Vascular, Abiomed, Medtronic, Terumo and Daiichi Sankyo. L. Paraggio received speaker fees from Abiomed and Terumo. F. Burzotta and C. Trani received speaker fees from Abbott Vascular, Abiomed, Medtronic, and Terumo. C. The other authors declare no conflicts of interest.

## Supporting information


**Supporting Figure S1:** Flow‐chart of patients selection and data extraction. **Supporting Figure S2a:** UpSet plot depicting the most frequent overlaps among different types of angiographic complications. **Supporting Figure S2b:** Rate of first‐occurring angiographic complications and subsequent complications. **Supporting Figure S3:** Common strategies adopted to address the angiographic complications. **Supporting Figure S4.:** Supporting Kaplan‐Meier survival curves with Log‐Rank tests. **Supporting Table S1:** Procedural details on angiographic complications. **Supporting Table S2:** Variables balance between PS‐matched cohorts. **Supporting Table S3:** Details on procedural outcome and survival data according to different first angiographic complication during the procedure.

## Data Availability

The data that support the findings of this study are available from the corresponding author upon reasonable request.
