# Supplementary material for: Clinical Impact of Angiographic Complications Occurring During Percutaneous Coronary Interventions
Source: Catheter Cardiovasc Interv. 2026 Feb 10;107(5):1573–83. doi: 10.1002/ccd.70515 (PMC13043793; doi:10.1002/ccd.70515)
Supplement: Supplementary file 1 — Supporting Figure S1: Flow‐chart of patients selection and data extraction. Supporting Figure S2a: UpSet plot depicting the most frequent overlaps among different types of angiographic complications. Supporting Figure S2b: Rate of first‐occurring angiographic complications and subsequent complications. Supporting Figure S3: Common strategies adopted to address the angiographic complications. Supporting Figure S4.: Supporting Kaplan‐Meier survival curves with Log‐Rank tests. Supporting Table S1: Procedural details on angiographic complications. Supporting Table S2: Variables balance between PS‐matched cohorts. Supporting Table S3: Details on procedural outcome and survival data according to different first angiographic complication during the procedure. [file CCD-107-1573-s001.docx]

**SUPPLEMENTARY MATERIAL**

**TABLE OF CONTENTS**

**Supplementary Method S1. Other angiographic definitions.**

**Supplementary Method S2. List of variables used for propensity score calculation.**

**Supplementary Figure S1. Flow-chart of patients selection and data extraction.**

**Supplementary Figure S2a. UpSet plot depicting the most frequent overlaps among different types of angiographic complications.**

**Supplementary Figure S2b. Rate of first-occurring angiographic complications and subsequent complications.**

**Supplementary Figure S3. Common strategies adopted to address the angiographic complications.**

**Supplementary Figure S4. Supplementary Kaplan-Maier survival curves with Log-Rank tests.**

**Supplementary Table S1. Procedural details on angiographic complications.**

**Supplementary Table S2. Variables balance between PS-matched cohorts.**

**Supplementary Table S3. Details on procedural outcome and survival data according to different first angiographic complication during the procedure.**

**Supplementary Method S1. Other angiographic definitions.**

A bifurcation lesion was defined according to the European Bifurcation Club as a coronary artery narrowing occurring adjacent to, and/or involving, the origin of a significant SB (1).

Severe lesion calcification was defined as radiopacities visible without cardiac motion before contrast injection, involving both sides of the arterial lumen (2,3).

The invasive British Cardiovascular Intervention Society Jeopardy Score (iBCIS-JS) was used as a simple angiographic scoring system to quantify the extent of jeopardized myocardium related to clinically significant CAD (4).

Angiographic success was defined as the achievement of a final TIMI grade 3 flow in all vessels, without persistent distal segment or SB flow impairment, and no devices entrapped, fractured, or left in the coronary circulation.

the incidence, management, and outcomes of angiographic complications by type and severity during non‐urgent, non‐CTO PCIs

**Supplementary Method S2. List of variables used for propensity score calculation.**

Logistic EUROscore, age, sex, prior PCI, prior CABG, Prior MI (> 3 months), clinical presentation (CCS vs stabilized staged ACS), prior HF hospitalization, peripheral artery disease, carotid artery disease, prior TIA/Stroke, diabetes on oral medication, diabetes on insulin therapy, family history of CAD, current smoker, former smoker (>1 year), dyslipidemia, hypertension, BMI>30, dialysis, radial access, single vessel disease, two vessels disease, three vessels disease, LM PCI, severely calcified lesion, bifurcation lesion, use of IVI.

**Supplementary Figure S1. Flow-chart of patients selection and data extraction.**

**
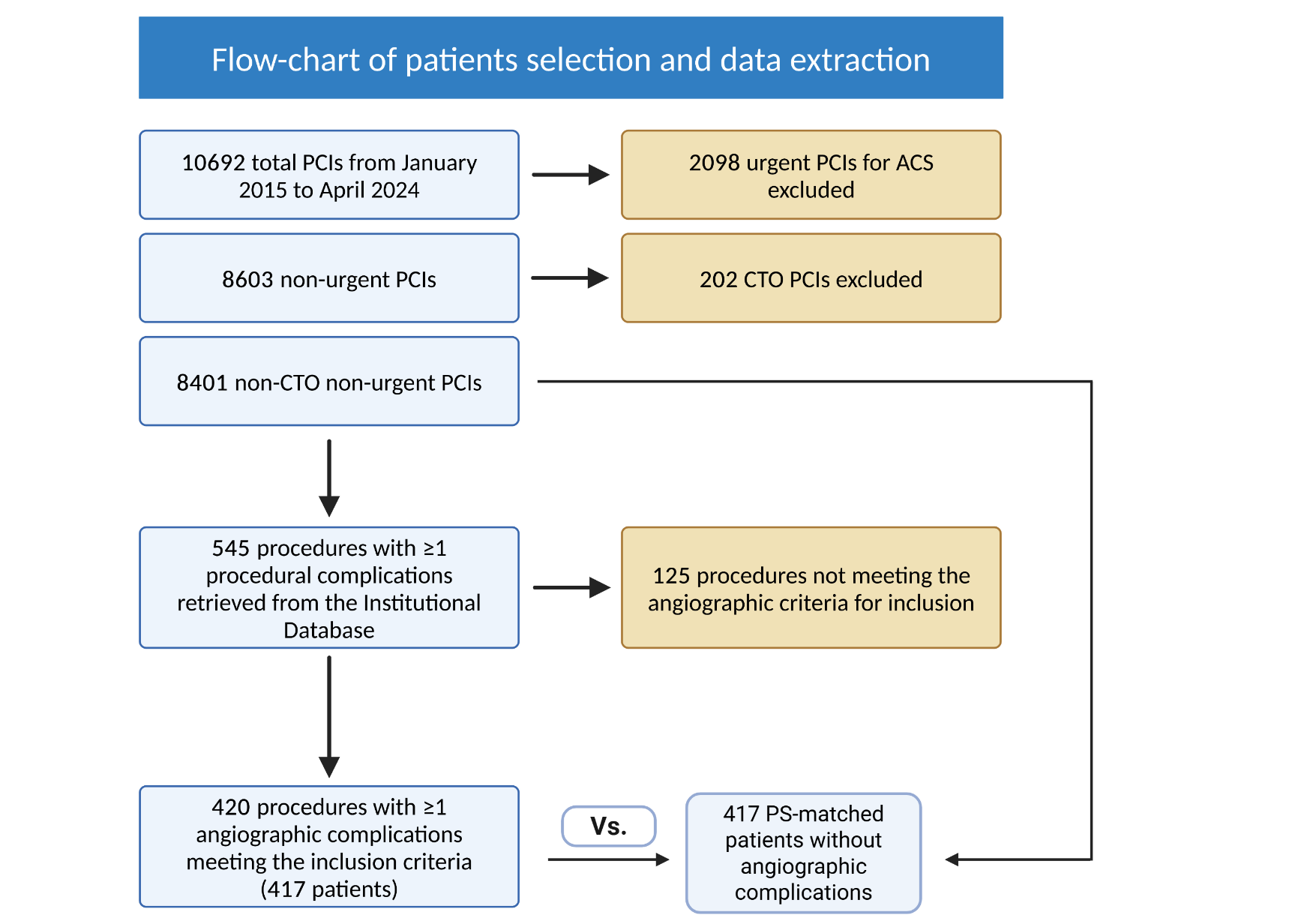
**

**Abbreviations:** ACS, acute coronary syndrome; CTO, chronic total occlusion; PCI, percutaneous coronary intervention; PS, propensity score.

**Supplementary Figure S2a. UpSet plot depicting the most frequent overlaps among different types of angiographic complications.** Each symbol represents a specific color-coded angiographic complication. The columns displaying connected symbols indicate groups of patients who experienced more than one specific angiographic complication during the index procedure. The numbers above the light blue columns show the number of patients in each group.

**Supplementary Figure 2b. Rate of first-occurring angiographic complications and subsequent complications.**

**
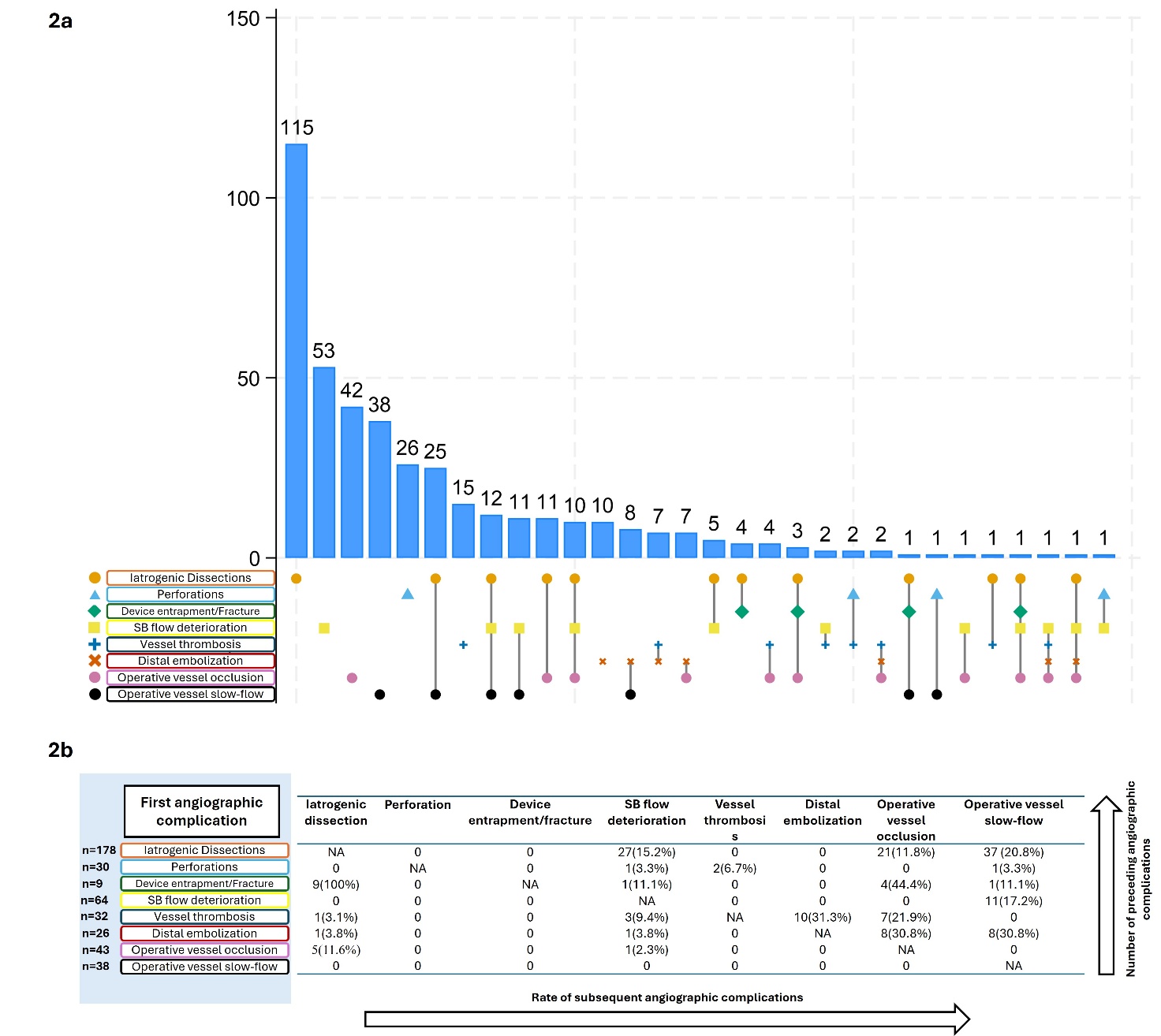
**

**Abbreviations**: SB, side branch.

**
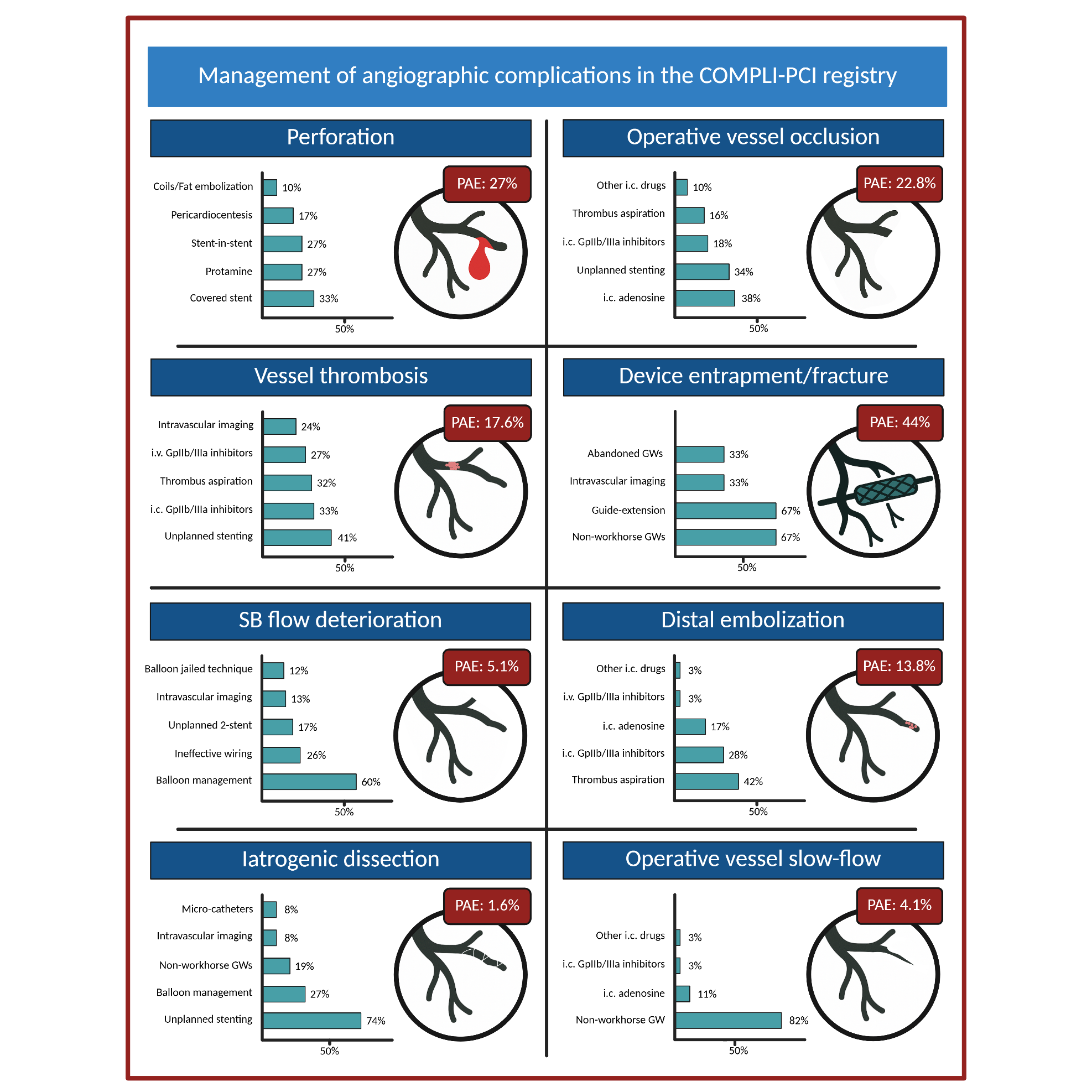
Supplementary Figure S3. Common strategies adopted to address angiographic complication in the registry.**

**Abbreviations**: Gp, Glycoprotein; GW, guidewires; PAE, procedural adverse events; SB, side-branch.

**Supplementary Figure S4. Supplementary Kaplan-Maier survival curves with Log-Rank tests.**

**a.** Kaplan-Maier survival curves with Log-Rank test between patients with or without complete final angiographic success.

**b.** Kaplan-Maier survival curves with Log-Rank test between patients with or without PAE following the angiographic complication.

**
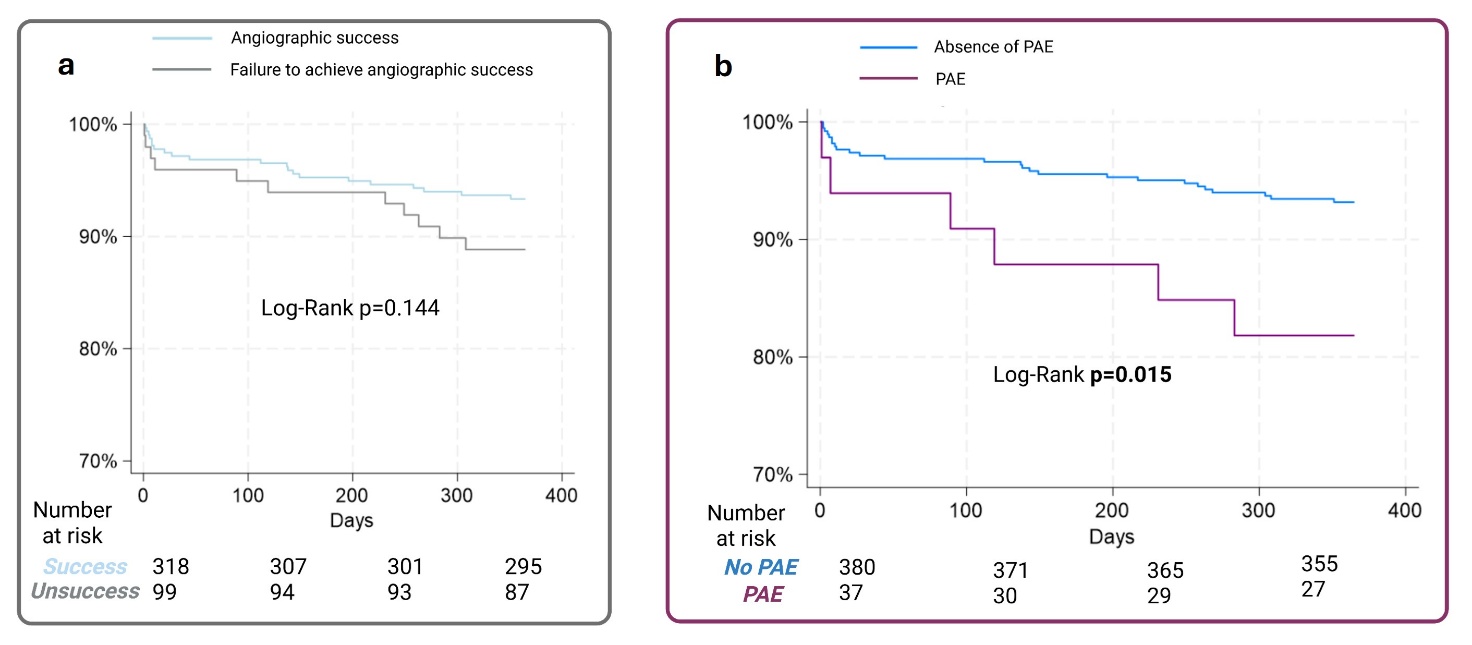
**

**Abbreviations**: PAE, procedural adverse events.

**Supplementary Table S1. Procedural details on angiographic complications.**

| **Type of angiographic complication** | **Mechanism** | **Type** | **Location** |
| --- | --- | --- | --- |
| **Iatrogenic dissection** | Catheter-induced: 2/189  Guidewire-induced:14/189  Pre-dilation-induced: 39/189  Stent-induced: 42/189  Post-dilation induced: 85/189 | **NHLBI classification**  A: 36/189  B: 60/189  C: 28/189  D: 32/189  E: 9/189  F: 25/189 | LM: 28/189  LAD: 112/189  LCX: 56/189  RCA: 30/189  SVG: 0/189 |
| **Perforations** | Guidewire-induced:  8/30  Balloon-induced:  17/30  Stent-induced:  5/30 | **Ellis Type**  I: 6/30  II: 11/30  III: 13/30 | LM: 2/30  LAD: 19/30  LCX: 3/30  RCA: 7/30  SVG: 1/30 |
| **SB flow deterioration** | During wiring: 3/98  After Predilation:19/98  After stent deployment:30/98  After post-dilation: 46/98  After “POT”: 22/98  After “Kissing”: 15/98 | Residual flow impairment: 42/98 | During LM PCI: 11/98  During LAD PCI: 76/98  During LCX PCI: 21/98  During RCA PCI: 9/98 |
| **Vessel thrombosis** | During wiring: 3/34  After pre-dilation: 4/34  After calcium debulking: 1/34  After stent deployment: 6/34  After post-dilation: 20/34  After “Kissing”: 9/34 | SB involvement: 6/34  Residual flow impairment: 6/34  Residual thrombus at the end of the procedure: 14/34 | LM: 8/34  LAD: 18/34  LCX: 13/34  RCA: 9/34  SVG: 2/34 |
| **Operative vessel occlusion** | During wiring: 8/83  After pre-dilation: 28/83  After calcium debulking: 4/83  After stent deployment: 21/83  After post-dilation: 23/83  After “kissing”: 4/83 | SB involvement: 24/83  Residual flow impairment: 27/83 | LM: 10/83  LAD: 45/83  LCX: 22/83  RCA: 22/83  SVG: 3/83 |
| **Distal embolization** | During wiring: 4/36  After pre-dilation: 8/36  After calcium debulking: 1/36  After stent deployment: 5/36  After post-dilation: 18/36  After “kissing”: 5/36 | SB involvement: 14/36  Residual flow impairment: 15/36 | LM: 5/36  LAD: 16/36  LCX: 12/36  RCA: 10/36  SVG: 2/36 |
| **Operative vessel slow-flow (TIMI 1-2)** | During wiring: 8/96  After pre-dilation: 19/96  After stent deployment: 28/96  After post-dilation: 42/96  After “kissing”: 13/96 | Residual flow impairment: 26/91 | LM: 5/91  LAD: 51/91  LCX: 26/91  RCA: 18/91  SVG: 2/91 |
| **Device entrapment/fracture** | Guidewire entrapment: 3/9  Guidewire fracture: 3/9  Stent/balloon crush/rupture: 5/9  Rotablator entrapment: 1/9  Microcatheter entrapment: 1/9 | Stent crush: 2/9  Fractured guidewire left behind: 3/9  Procedural flow deterioration: 5/9 | LAD: 3/9  LCX: 2/9  RCA: 4/9 |

**Abbreviations:** LAD, left anterior descending; LCX, left circumflex; LM, left main; PCI, percutaneous coronary intervention; RCA, right coronary artery; SB, side-branch; SVG, saphenous vein graft; TIMI, thrombolysis in myocardial infarction.

**Supplementary Table S2. Variables balance between PS-matched cohorts.**

| **Variable** | **Patients with angiographic complications (n=417)** | **Patients without angiographic complications (n=417)** | **Standardized mean difference** |
| --- | --- | --- | --- |
| **Age** | 70.8 ± 10.3 | 71.3±9.7 | -0.059 |
| **Sex (male)** | 304 (72.4) | 307 (73.6) | -0.026 |
| **BMI > 30** | 51 (12.1) | 52 (12.4) | -0.014 |
| **Hypertension** | 320 (76.7) | 327 (78.4) | -0.040 |
| **Diabetes**  **On oral medications**  **On insulin therapy** | 77(18.4)  48 (11.5) | 73 (17.5)  50 (11.9) | 0.024  -0.014 |
| **Current smoker** | 62 (14.8) | 55 (13.1) | 0.047 |
| **Former smoker** | 91 (21.8) | 83 (19.9) | 0.048 |
| **Dyslipidemia** | 258 (61.8) | 245 (58.7) | 0.063 |
| **Family history of CAD** | 84 (20.1) | 83 (19.9) | 0.005 |
| **Creatinine > 1.2 mg/dL** | 21 (5.0) | 17 (4.0) | 0.045 |
| **Dialysis** | 9 (2.1) | 14 (3.3) | -0.073 |
| **Logistic EUROscore** | 3.4 ± 4.2 | 3.8 ± 5.9 | -0.079 |
| **Prior MI (> 3 months)** | 37 (8.8) | 43 (10.3) | -0.048 |
| **Prior PCI** | 140 (33.6) | 153 (36.7) | -0.065 |
| **Prior CABG** | 39 (9.3) | 32 (7.6) | 0.060 |
| **Prior HF hospitalization** | 40 (9.5) | 28 (6.7) | 0.105 |
| **Peripheral artery disease** | 20 (4.8) | 24 (5.7) | -0.042 |
| **Carotid artery disease** | 22 (5.2) | 18 (4.3) | 0.044 |
| **Prior TIA/stroke** | 7 (1.7) | 5 (1.2) | 0.040 |
| **Clinical presentation:**  **CCS**  **Staged PCI after ACS** | 355 (85.1)  62 (14.9) | 364 (87.3)  53 (12.7) | 0.062 |
| **Single-vessel disease** | 177 (42.5) | 184 (44.1) | -0.053 |
| **Two-vessels disease** | 151 (36.2) | 157 (37.7) | -0.034 |
| **Three vessels disease** | 89 (21.3) | 76 (18.2) | 0.073 |
| **Severely calcified lesion** | 103 (24.7) | 99 (23.7) | 0.022 |
| **Bifurcation lesion** | 175 (41.9) | 165 (39.6) | 0.048 |
| **LM PCI** | 34 (8.1) | 33 (7.9) | 0.008 |
| **Radial access use** | 401 (96.1) | 392 (94.0) | -0.150 |
| **Use of IVI** | 42 (10.0) | 42 (10.0) | 0.000 |
|  |  |  |  |

**Abbreviations:** ACS, acute coronary syndrome; CABG, coronary artery bypass grafting; CAD, coronary artery disease; CCS, chronic coronary syndrome; HF, heart failure; MI, myocardial infarction; IVI, intravascular imaging; PCI, percutaneous coronary intervention; PS, propensity score; TIA, transient ischemic attack.

**Supplementary Table S3. Details on procedural outcome and survival data according to different first angiographic complication during the procedure.**

| **Type of first angiographic complication** | **PAE** | **30-days death** | **1-year death** |
| --- | --- | --- | --- |
| **Iatrogenic dissection** | 2/178 (1.1%) | 4/178 (2.2%) | 12/178 (6.7%) |
| **Perforation** | 8/30 (26.6%) | 4/30 (13%) | 5/30 (16%) |
| **Device entrapment/fracture** | 4/9 | 0/9 | 1/9 |
| **SB flow deterioration** | 2/64 (3.1%) | 1/64 (1.6%) | 2/64 (3.1%) |
| **Vessel thrombosis** | 5/32 (15.6%) | 2/32 (6.3%) | 2/32 (6.3%) |
| **Distal embolization** | 3/26 (11.5%) | 0/26 | 1/26 (3.8%) |
| **Operative vessel occlusion** | 11/43 (25.6%) | 5/43 (11.6%) | 11/43(25.6%) |
| **Operative vessel slow-flow (TIMI 1-2)** | 2/38 (5.3%) | 1/38 (2.6%) | 2/38 (5.3%) |

*Values are n (%)*

**Abbreviations:** CI, confidence interval; HR, hazard ratio; OR, odds ratio; PAE, procedural adverse event; SB, side-branch; TIMI, thrombolysis in myocardial infarction.

**REFERENCES**

1. Louvard Y., Medina A. Definitions and classifications of bifurcation lesions and treatment. EuroIntervention 2015;11(V):V23–6. Doi: 10.4244/EIJV11SVA5.

2. Généreux P., Madhavan MV., Mintz GS., et al. Ischemic Outcomes After Coronary Intervention of Calcified Vessels in Acute Coronary Syndromes. J Am Coll Cardiol 2014;63(18):1845–54. Doi: 10.1016/j.jacc.2014.01.034.

3. Kawashima H., Serruys PW., Hara H., et al. 10-Year All-Cause Mortality Following Percutaneous or Surgical Revascularization in Patients With Heavy Calcification. JACC Cardiovasc Interv 2022;15(2):193–204. Doi: 10.1016/j.jcin.2021.10.026.

4. De Silva K., Morton G., Sicard P., et al. Prognostic Utility of BCIS Myocardial Jeopardy Score for Classification of Coronary Disease Burden and Completeness of Revascularization. Am J Cardiol 2013;111(2):172–7. Doi: 10.1016/j.amjcard.2012.09.012.
